# Associations between physical activity and brain structure in a large community cohort

**DOI:** 10.1038/s41598-025-04010-7

**Published:** 2025-05-29

**Authors:** Alexandra La Hood, Chris Moran, Stephanie Than, Alicia Lu, Taya A. Collyer, Richard Beare, Velandai Srikanth

**Affiliations:** 1 Peninsula Clinical School, School of Translational Medicine, Monash University, PO Box 52, Frankston, VIC 3199 Australia; 2 Department of Geriatric Medicine, Peninsula Health, 24 Separation Street, Mornington, VIC 3931 Australia; 3 National Centre for Healthy Ageing, PO Box 52, Frankston, VIC 3199 Australia; 4 School of Public Health and Preventive Medicine, Monash University, 553 St Kilda Road, Melbourne, VIC 3004 Australia; 5 Department of Home, Acute and Community, Alfred Health, 260 Kooyong Rd, Caulfield, VIC 3162 Australia; 6 Department of Geriatric Medicine, Western Health, 160 Gordon Street, Footscray, VIC 3011 Australia; 7 Developmental Imaging, Murdoch Children’s Research Institute, 50 Flemington Rd, Parkville, Melbourne, VIC 3052 Australia

**Keywords:** Exercise, Body mass index, Brain volume, Accelerometer, Cognitive ageing, Magnetic resonance imaging, Imaging, Ageing, Risk factors

## Abstract

Low physical activity (PA) and obesity are dementia risk factors, but it is unclear whether the association between PA and brain health is dependent upon degree of obesity. We aimed to examine the association between PA and brain structure and explore whether it varies by body mass index (BMI). We examined the associations between accelerometer-measured PA and magnetic resonance imaging-measured brain structure using cross-sectional data from a population-based cohort of people in midlife (UK Biobank). Using regression modelling, we explored whether these associations were mediated by BMI or other cardiometabolic risk factors. Complete data were available for 16,725 participants (median age 65 years, 55% women, median BMI 26). Greater PA was positively associated with total brain (β = 3.67, *p* < 0.0001), grey matter (β = 3.12, *p* < 0.0001), white matter (β = 0.43, *p* < 0.0001) and total hippocampal volumes (β = 0.05, *p* < 0.0001), and inversely associated with white matter hyperintensity volume (β = − 4.6 × 10^−5^, *p* < 0.0001). Although there was no interaction between PA and BMI in explaining brain volumes, BMI attenuated the PA-brain volume associations by between 18 and 58%. The PA-brain volume associations were further attenuated by other cardiometabolic risk factors and in the case of grey matter and hippocampal volume, was no longer statistically significant in fully adjusted models. Greater PA is associated with a healthier profile in brain volumes. BMI and cardiometabolic factors mediated the relationship between PA and grey matter and hippocampal volume and partially mediated its relationship with other brain volumes. These results support a mechanistic basis for PA in optimizing cardiometabolic risk for dementia prevention.

**Supplementary Information:**

The online version contains supplementary material available at 10.1038/s41598-025-04010-7.

## Introduction

Greater levels of physical activity (PA) in midlife and older age are associated with lower dementia risk^1–4^, presumably through its favourable impact on cognitive performance^5,6^ and brain structural measures such as, grey matter^7–10^ total brain^11,12^ and white matter volumes^13–15^.

Prior work examining the links between PA and brain health have either not considered the role of obesity or have considered its effect as a fixed constant. Obesity is associated with an increased risk of dementia, poorer cognition, and poorer brain structure in older adults with a higher body mass index (BMI)^6,16–19^. People who are overweight and obese in midlife carry the highest risk of cognitive decline^16^. However, recent work has reported that the degree of obesity in older adults may determine the extent to which PA contributes to brain structures such as grey matter volume^20^. This relationship between PA, obesity and brain structure may commence earlier in life, with a recent longitudinal study demonstrating obesity and poor cardiorespiratory fitness in childhood were found to be associated with poorer cognition in midlife^21^.

Current consensus guidelines recommend regular PA and maintaining a healthy BMI to promote brain health^22–24^, particularly during mid-life because the pathological changes of dementia often appear decades prior to the onset of symptoms^25^. However, it is unclear whether the beneficial associations between PA and brain health are related to the direct impact of PA on the brain, or whether obesity plays a role. As such, it is not clear whether certain groups benefit more from PA depending on BMI, or whether PA itself is more relevant to brain health.

We therefore aimed to examine the association between PA and magnetic resonance imaging (MRI) measures of brain volumes in people in mid-later life and explore whether these associations were altered by the degree of obesity in a large, community-dwelling sample of people based in the United Kingdom.

## Methods

### Study participants and data

Data used in this study was sourced from the UK Biobank (http://www.ukbiobank.ac.uk/), a large open-access database available internationally. The primary aim of the UK Biobank is to provide data to researchers that will ultimately improve disease prevention, diagnosis, and management in middle and older age. Complete recruitment and UK Biobank procedural details are available on www.ukbiobank.ac.uk and have been previously published^26^. Recruitment of 503,325 volunteers aged 40–69 years from the United Kingdom occurred between 2006 and 2010. Participants were eligible if they were registered with the National Health Service and resided within travelling distance of 22 assessment centres.

### Clinical and genetic data

Demographic data was collected through detailed touchscreen questionnaires, nurse-led interviews, and online surveys. Covariables self-reported in this study included ethnicity, education level, smoking status, frequency of alcohol consumption and mood (whether depressed mood was experienced in the last 2 weeks). Socioeconomic status was identified using the Townsend deprivation index^27^. Composite variables were formulated for hypertension, ischaemic heart disease, hypercholesterolaemia, stroke and diabetes, combining self-reported diagnoses, relevant medication history and ICD-10 codes as per previously published UK Biobank work^28^. Self-reported PA was collected using the validated International Physical Activity Questionnaire (IPAQ) tool, which classified participants into three groups: low (in the low category or activity levels not meeting moderate or high criteria), moderate (meeting one of three criteria, ≥ 3 days of vigorous activity, ≥ 20 min per day, ≥ 5 days of moderate-intensity activity and/or ≥ 30 min of walking per day or ≥ 5 days of any combination of activity meeting a minimum total of 600 MET-minutes/week) and high (≥ 3 days of vigorous intensity activity achieving a minimum total of 1500 MET-minutes/week or ≥ 7 days of any combination of activity meeting a minimum total of 3000 MET-minutes/week)^29,30^. Demographic, health and physical examination were collected at the time of the MRI visit. Anthropometric measures were taken by trained clinic research staff. A body composition analyser was used to measure weight (Tanita BC 418; Tanita Corporation, Arlington Heights, Ill). A wall-mounted height measure was used to measure height (Seca 240; Seca, Hamburg, Germany). BMI calculation used the standard formula of weight (kilograms) divided by height (metres) squared. Biological samples were taken for genetic analysis for Apolipoprotein E (*APOE*) *ε*4 carrier status, a dementia risk factor. Genotyping was carried out through the UK BiLEVE array or UK Biobank Axiom array. Participants carrying at least one *APOE ε*4 allele were deemed *APOE ε*4 positive.

### Brain volume measurement

Between March 2014 and January 2018, 100,000 participants of the original cohort were invited back to undergo an MRI. A 3T MRI imager (Version VD13A SP4, Siemens Skyra, Siemens Healthcare, Erlangen, Germany) with a standard 32-channel radiofrequency receiver head coil was used to scan the brain. The UK Biobank specific MRI acquisition protocol and imaging derived phenotypes (IDP) generation pipeline has been previously published^31^. Brain tissue volumes were normalised for head size using the estimated skull external surface from T1 imaging and SIENAX analysis generating both normalised and non-normalised imaging-derived phenotypes^32^. T1-weighted and T2-weighted fluid attenuated inversion recovery scans from the following IDPs were used in this study: total and regional grey matter volume (GMV), white matter volume (WMV) and total brain (GMV + WMV) volume (TBV). Total hippocampal volume (THV) was calculated using FMRIB’s Integrate Registration and Segmentation Tool (FIRST). T1 and T2 FLAIR imaging was used to measure white matter hyperintensity volume (WMHV).

### Physical activity measurement

An email invitation to wear an accelerometer for 7 days was sent to 236,519 participants with a valid email address, and an Axivity AX3 wrist-worn triaxial accelerometer was mailed to all participants on acceptance of the email invitation. Data collection for 103,712 participants occurred between June 2013 and January 2016. Accelerometer protocol details for data extraction and processing have previously been outlined^33^. Participants were instructed to wear the device continuously for 7 days, which was programmed to start functioning two working days after postage at 10:00 am. Acceleration data was collected over a 7-day period at 100 Hz with a dynamic range of ± 8* g* and summarised as the overall acceleration average, which is the mean vector magnitude in milli-gravity units (m*g*). We use overall acceleration average as our primary measure for PA. Only data with good wear time (at least 72 h of data and having data in each 1-h period of the 24-h cycle) and good calibration was included in our study. Data was downloaded from the UK Biobank Application Management System in September 2022.

### Ethics

All participants provided written consent with details accessible through http://www.ukbiobank.ac.uk/. The UK Biobank approved this study (Project ID 24954). Ethics approval was granted for this study by the National Research Ethics Service Committee (reference 11/NW/0382) and Monash University Human Research Ethics Committee (project ID: 18734).

### Statistical analysis

Descriptive summary statistics were used to examine the sample, expressed as a median (interquartile range [IQR]) for continuous variables or number (percentage) for categorical variables. Only participants with complete cardiometabolic data, brain MRI measures and accelerometer data were included. Associations between PA (overall acceleration average) and measures of brain structure, including TBV, GMV, WMV, THV and WMHV were examined using linear regression. The distribution of the model residuals were examined using QQ plots. Outcome measures in models with non-normally distributed residuals underwent testing of a number of different transformations to identify the best transformation to meet regression modelling assumptions. Recent work has reported non-linear associations between accelerometer-based measures and cognitive function in the UK Biobank^34^. We therefore examined the presence of non-linear associations between PA and each of the brain measures (up to quartic higher orders) and where present, these were included in the final models. Where indicated, the unstandardised beta coefficients from the univariable model were then adjusted for an age × sex interaction identified in previous UK Biobank studies^28^. We then tested for the presence of an interaction product term *PA* × *BMI*, where BMI was used as a continuous variable*.* We additionally examined for between-BMI strata^35^ differences in the associations between PA and each of the brain regions in age, sex and fully adjusted models. The percentage change from the beta coefficient of PA was calculated between relevant models. We then examined the relationship between PA and brain volumes in models fully adjusted for potential sociodemographic and cardiometabolic confounders.

We also performed a number of sensitivity analyses. To explore the role of waist circumference rather than BMI, we repeated our preliminary models replacing BMI with waist circumference as a potential mediator of relationship between PA and brain volumes. We also categorised individual’s objectively-measured physical activity over the collection period into three broad activity groups using established cut-points (sedentary: mean < 45 mg, light: mean 45–100 mg and moderate > 100 mg)^36,37^. We then examined for the presence of differences in brain volumes between these groups in age and sex-adjusted models. We additionally explored the correlation between subjectively reported physical activity (using the IPAQ tool) and objectively-measured physical activity. This was then followed by an analysis of the association between subjectively reported physical activity and brain volumes. Given there is correlation between the five brain structure outcomes of interest, Bonferroni correction was used to adjust for multiple comparisons (corrected *p* = 0.01). All analyses were performed using R (version 4.2.1) in RStudio (version 2023.06.0 Build 421).

## Results

### Sample characteristics

Complete PA and brain volumetric data were available for 16,725 participants with 486,600 volunteers excluded due to missing data. Sample characteristics are described in Table 1. The median age of the sample was 64.9 years (IQR 11.9 years). Approximately 55% of participants were women and almost 98% reported being of white ethnicity. The proportion of *APOE ε*4 carriers was 27.6%. Most participants were current consumers of alcohol (94%) and had a history of smoking (53%). Over a third (37%) of the sample had a history of hypertension. The median BMI of the sample was 25.7 (IQR 5.3).

**Table 1:** Sample characteristics.

	Whole group
	N (%) or median ± IQR
Number of participants	16,725
Demographics	
Age (years)	64.9 ± 11.9
Female	9192 (55.0)
Townsend deprivation index	− 2.63 ± 3.37
White ethnicity	16,260 (97.5)
Tertiary degree	8302 (53.0)
Smoking status	
Current or previous smoker	8853 (53.4)
Never smoked	7730 (46.6)
Alcohol status	
Current or previous alcohol	15,544 (93.5)
Never drank alcohol	528 (3.2)
Sleep duration (hours per day)	7 ± 1
Depressed mood in last 2 weeks	2810 (16.9)
*APOE ε*4 carrier	4510 (27.6)
Comorbidities	
Diabetes mellitus	876 (5.2)
Ischaemic heart disease	847 (5.1)
Hypertension	6149 (36.8)
Hypercholesterolaemia	3963 (23.7)
Stroke	594 (3.6)
Physical characteristics	
Body mass index (kg/m^2^)	25.7 ± 5.3
< 18.5	141 (0.8)
18.5–25	6826 (40.8)
25–30	6435 (39.8)
> 30	2772 (17.1)
Waist circumference (cm)	87 ± 18
Hip circumference (cm)	100 ± 10
Systolic BP (mmHg)	139 ± 27
Diastolic BP (mmHg)	78 ± 15

Table 2 describes the physical activity and MRI brain volumes of participants. The median accelerometer wear time was 6.9 days (IQR 0.3 days). The median overall acceleration average was 27.6 mg (IQR 9.9 mg). Approximately 20% of people self-reported low physical activity. The median total brain volume of participants was 1495 cm^3^ (IQR 102 cm^3^).

**Table 2:** Physical activity and brain volumes of participants.

	Whole group
	N (%) or median ± (IQR)
Physical activity	
Summed MET minutes per week for all activity (mins)	1744 ± 2425
IPAQ activity group	
Low	2598 (18.1)
Moderate	5983 (41.8)
High	5738 (40.1)
Accelerometer wear time (days)	6.9 (0.3)
Acceleration average (m*g*)	27.6 (9.9)
Brain volumes (cm^3^)	
Total brain volume	1495.3 ± 102.2
Grey matter volume	792.9 ± 65.3
White matter volume	701.4 ± 55.7
White matter hyperintensities	3.6 ± 5.3
Total hippocampal volume	10.0 ± 1.5

*MET* metabolic equivalent task, *IPAQ* international physical activity questionnaire.

### Physical activity, BMI and brain volumes

#### Total brain volume (TBV)

Table 3 presents the associations between PA and TBV. In addition to a linear association between greater PA and greater TBV (β = 3.67, 95% CI 2.82–4.51, *p* < 2 × 10^−16^) we found statistically significant quadratic (*p* = 2 × 10^−11^) and cubic (*p* = 0.01) terms. We confirmed our previously published age × sex interaction on TBV (β = − 0.79, 95% CI − 1.03 to − 0.56, *p* = 7 × 10^−11^)^28^. The association between PA and TBV persisted when this age × sex interaction was included in the model (β = 1.46, 95% CI 0.77–2.15, *p* = 3.4 × 10^−5^). We did not find an interaction between PA and BMI on TBV (β = − 0.007, 95% CI − 0.03 to 0.02, *p* = 0.63). The association between PA and TBV was attenuated by 25% when BMI was included in the model (β = 1.09, 95% CI 0.38–1.80, *p* = 0.002). The association between PA and TBV was no longer statistically significant when adjusted for other cardiometabolic risk factors (β = 0.72, 95% CI − 0.02 to 1.47, *p* = 0.06). The association between PA and TBV was similar across BMI strata (Supplementary Table S1). Substituting waist circumference for BMI resulted in a similar pattern of results with the addition of waist circumference attenuating the association between PA and TBV volume by 35% (Supplementary Table S2).

**Table 3:** Influence of body mass index on associations between physical activity and brain volumes.

	Total brain volume^a^ β-coefficient (95% CI) *p* value	Grey matter volume^a^ β-coefficient (95% CI) *p* value	White matter volume β-coefficient (95% CI) *p* value	Total hippocampal volume^a^ β-coefficient (95% CI) *p* value	White matter hyperintensity volume^a,b^ β-coefficient (95% CI) *p* value
Model 1					
Physical activity	3.67 (2.82 to 4.51) < 2 × 10^−16^	3.12 (2.57 to 3.67) < 2 × 10^−16^	0.43 (0.36 to 0.51) < 2 × 10^−16^	0.05 (0.03 to 0.06) 1 × 10^−11^	− 4.6 × 10^−5^ (− 6 × 10^−5^ to − 3 × 10^−5^) 3 × 10^−15^
Model 2					
Physical activity + (age × sex)	1.46 (0.77 to 2.15) 3.4 × 10^−5^	1.19 (0.77 to 1.60) 2.0 × 10^−8^	0.11 (0.03 to 0.18) 0.004	0.015 (0.002 to 0.03) 0.02	− 2.6 × 10^−5^ (− 4 × 10^−5^ to − 2 × 10^−5^) 3 × 10^−7^
Model 3					
Physical activity + BMI + (age × sex)	1.09 (0.38 to 1.80) 0.002	0.76 (0.34 to 1.18) 0.0004	0.13 (0.06 to 0.21) 0.0006	0.012 (− 0.001 to 0.02) 0.07	− 1.6 × 10^−5^ (− 3 × 10^−5^ to − 6 × 10^−6^) 0.002
Change in beta compared with Model 2	↓25%	↓36%	↑18%	↓20%	↓38%
Model 4					
(Physical activity × BMI) + (age × sex)	− 0.007 (− 0.03 to 0.02) 0.63	0.01 (− 0.004 to 0.03) 0.13	− 0.01 (− 0.03 to 0.003) 0.11	0.0001 (− 0.006 to 0.0004) 0.61	− 4.0 × 10^−8^ (− 4 × 10^−7^ to − 4 × 10^−7^) 0.85
Model 5					
Physical activity + (age × sex) + BMI + Fully adjusted for listed covariables^c^	0.72 (− 0.02 to 1.47) 0.06	0.42 (− 0.02 to 0.87) 0.06	0.14 (0.06 to 0.22) 0.0009	0.008 (− 0.005 to 0.02) 0.24	− 7.3 × 10^−6^ (− 2 × 10^−5^ to 3 × 10^−6^) 0.18

*BMI* body mass index, *CI* confidence interval.

^a^Models contain quadratic and cubic physical activity terms.

^b^Log-transformed.

^c^Fully adjusted model includes ethnicity + Townsend deprivation index + hypertension + ischaemic heart disease + hypercholesterolaemia + stroke + diabetes + Apolipoprotein E ε4 + smoking + education + sleep + alcohol + mood.

#### Grey matter volume (GMV)

Greater PA was associated with greater GMV (β = 3.12, 95% CI 2.57–3.67, *p* < 2 × 10^−16^) (Table 3) and included statistically significant quadratic (*p* = 2 × 10^−16^) and cubic (*p* = 2 × 10^−5^) terms. We confirmed the previously published age × sex interaction on GMV (β = − 0.50, 95% CI − 0.65 to − 0.36, 5 × 10^−12^)^28^. The association between PA and GMV remained when age × sex was added to the model (β = 1.19, 95% CI 0.77–1.60, *p* = 2 × 10^−8^). We did not find a statistically significant PA × BMI interaction on GMV (*p* = 0.13). The addition of BMI attenuated the association between PA and GMV by 36% (β = 0.76, 95% CI 0.34–1.18, *p* = 0.0004). The association between PA and GMV was no longer statistically significant when adjusted for other cardiometabolic risk factors (β = 0.42, 95% CI − 0.02 to 0.87, *p* = 0.06). The association between PA and GMV was similar across BMI strata (Supplementary Table S1). Substituting waist circumference for BMI resulted in a similar pattern of results with the addition of waist circumference attenuating the association between PA and GMV volume by 40% (Supplementary Table S2).

#### White matter volume (WMV)

The association between PA and WMV is presented in Table 3. Greater PA was associated with greater WMV (β = 0.43, 95% CI 0.36–0.51, *p* < 2 × 10^−16^) and there were no statistically significant non-linear terms (all *p* > 0.06). An age × sex interaction was confirmed (β = − 0.29, 95% CI − 0.45 to − 0.13, *p* = 0.0003)^28^, and the association between PA and WMV remained significant after accounting for this interaction (β = 0.11, 95% CI 0.03–0.18, *p* = 0.004). There was no interaction between PA × BMI on WMV (*p* = 0.11). With the addition of BMI, the association between PA and WMV increased by 18% (β = 0.13, 95% CI 0.06–0.21, *p* = 0.0006), and was minimally altered when adjusted for other cardiometabolic risk factors (β = 0.14, 95% CI 0.06–0.22, *p* = 0.0009). The association between PA and WMV was similar across BMI strata (Supplementary Table S1). Substituting waist circumference for BMI resulted in a similar pattern of results with the exception that the addition of waist circumference attenuated the association between PA and WMV volume by 9% prior to the inclusion other cardiometabolic risk factors (Supplementary Table S2).

#### Total hippocampal volume (THV)

Greater PA was associated with greater THV (β = 0.05, 95% CI 0.03–0.06, *p* = 1 × 10^−11^) (Table 3) and included statistically significant quadratic (*p* = 2 × 10^−6^) and cubic (*p* = 0.009) PA terms. We identified the known interaction between age × sex on THV (β = 0.015, 95% CI 0.01–0.02, *p* = 2 × 10^−12^)^28^. After correction for multiple comparisons, the association between PA and THV remained when the age × sex interaction was included in the model (β = 0.015, 95% CI 0.002–0.03, *p* = 0.02). We did not find an interaction between PA × BMI on THV (*p* = 0.61). The association between PA and THV was attenuated by 20% following the addition of BMI (β = 0.012, 95% CI − 0.001 to 0.02, *p* = 0.07). The association between PA and THV was similar across BMI strata (Supplementary Table S1). Similar to that seen with BMI, substituting waist circumference for BMI and adding this to the model of PA, age and sex (Supplementary Table S2) removed the association between PA and THV (*p* = 0.17).

#### White matter hyperintensity volume (WMHV)

The WMHV variable was log-transformed following review of distribution of model residuals. Greater PA was associated with lower WMHV (β = − 4.6 × 10^−5^, 95% CI − 6 × 10^−5^ to − 3 × 10^−5^, *p* = 3 × 10^−15^) (Table 3) and included statistically significant quadratic (*p* = 6 × 10^−5^) and cubic (*p* = 0.02) terms. We confirmed an age × sex interaction on WMHV (β = 1.2 × 10^−5^, *p* = − 2 × 10^−12^)^28^, and when age × sex was added to the model, the PA and WMHV association remained (β = − 2.6 × 10^−5^, 95% CI − 4 × 10^−5^ to − 2 × 10^−5^, *p* = 3 × 10^−7^). We did not find an interaction between PA × BMI on WMHV (*p* = 0.85). The association between greater PA and lower WMHV was reduced by 38% following the addition of BMI to the model (β = − 1.6 × 10^−5^, 95% CI − 3 × 10^−5^ to − 6 × 10^−6^, *p* = 0.002). The PA and lower WMHV association was no longer statistically significant following addition of the other cardiometabolic risk factors (*p* = 0.18). The association between PA and WMHV was similar across BMI strata (Supplementary Table S1). Substituting waist circumference for BMI resulted in a similar pattern of results (Supplementary Table S2).

### Further sensitivity analyses

To explore the role of intensity of physical activity, we examined for the presence of brain structural differences in those with generally sedentary, generally light and moderately vigorous measures over the period of accelerometer wear. In age and sex adjusted models, we found that those who were lightly vigorous in their general activity had greater THV (β = 0.12, *p* = 0.003) and lower WMHV (β = − 9 × 10^−5^, *p* = 0.01) than those generally sedentary. We did not find any other statistically significant association between physical activity intensity and any of the other brain volumes (Supplementary Table S3).

Self-reported physical activity data was available for 14,319 participants. There was a strong correlation between self-reported and objective physical activity (Spearman’s rank = 3 × 10^11^, *p* = 2.2 × 10^−16^). The median objective physical activity was lowest in those reporting low physical activity (25.1 mg) and was greatest in those reporting the greatest physical activity (29.6 mg) (Supplementary Table S4). The median regional brain volumes were similar across those self-reporting low, moderate and high levels of physical activity (Supplementary Table S4). The association between self-reported physical activity and each of the brain measures was similar across each of the low, moderate and high after Bonferroni correction for multiple comparisons (Supplementary Table S5).

## Discussion

In this large population-based study of people living in the UK, we found that greater levels of PA were associated with healthier brain volumes across multiple brain regions including TBV, GMV, WMV, and WMHV. Although BMI, waist circumference and cardiometabolic risk factors mediated most of these associations, greater PA remained independently associated with healthier TBV, WMV, and WMHV. Our results support existing evidence that physical activity is beneficial for brain health^22–24^ and suggest that its benefit may be indirect via cardiometabolic modulation, or direct via other mechanisms.

We found that the association between PA and most brain volumes was mediated by, but also independent of either BMI or waist circumference. It is well established that PA can improve BMI, waist circumference and cardiometabolic health^35,38,39^ and it is likely that some of the associations we see between greater PA and healthier brain measures reflect these processes^20,40^. Similarly, PA may contribute to better brain health by reducing sedentary time, as increased sedentary behaviour has been linked to a higher risk of dementia^3^. A cross-sectional study exploring accelerometer-measured step count, and MRI-measured brain structure found adults who performed lower intensity PA and had greater sedentary time gained the greatest PA-driven brain volume benefit^8^. It is possible PA may play a role in reducing duration of sedentary time, avoiding the mechanisms that drive the inflammatory state associated with the metabolic syndrome.

PA may also directly affect brain health outside of obesity and cardiovascular risk-related pathways. However, the exact mechanisms through which this occurs are unknown. PA appears to promote neuroprotective growth factors such as insulin-like growth factor-1 (IGF-1), brain-derived neurotrophic factor (BDNF) and vascular endothelial growth factor (VEGF)^41–43^. These factors have been shown to inhibit neuronal death^44^, stimulate neurogenesis^42,45^ and increase cerebral angiogenesis and capillary vascularity^43^ in non-human models. These effects may play an important role in maintaining or optimising brain volumes^44^. PA also promotes lactate production which stimulates cerebral angiogenesis, improves vasoreactivity and drives the utilisation of alternate energy sources such as ketones^46^. Improved vasoreactivity has been associated with lower dementia risk^47^, possibly through enhanced lymphatic clearance of amyloid^48^. Lactate-mediated pathways may also optimise cerebral blood flow and metabolic capacity by improving functional connectivity and energy efficiency, thus increasing the brain’s compensatory abilities^44^. As a potent driver of anti-inflammatory pathways, PA promotes the release of anti-inflammatory interleukins and surpresses pro-inflammatory cytokines, as well as reducing oxidative stress^49^ associated with an increased risk of all-cause dementia^50,51^.

This study has several strengths. The large sample size permits greater confidence in our estimates and enables the exploration of the role of a number of different relevant factors. By studying people in mid-later life, the focus is on a time in life in which there is greater interest in using exercise interventions to reduce dementia risk before clinical signs become apparent^22–24^. The availability of a wrist-worn objective measure of PA minimises recall bias than can occur with self-reported PA and has a greater adherence and better activity capture than waist-worn monitors^33,52^. Although we found good correlation between self-reported and objectively measured physical activity in our study, the absence of between-group differences in brain structure in self-reported physical activity may be related to the smaller number of observations available or the lack of granular detail compared to objective, accelerometer data. This study also has several limitations. As a cross-sectional study, we cannot make any claims regarding causation, although with ongoing UK Biobank data collection planned, longitudinal data will become available. PA activity was not collected at the same time as the MRI were performed (collected within 2 years), further limiting claims of causality and the study being classified as truly cross-sectional. Participants of the UK Biobank study are volunteers, with the possibility of recruitment bias based on being healthier, more health literate or more engaged in their own health. Indeed, a study comparing UK Biobank participants to the general UK population found they were healthier, more educated and living in areas with a greater socioeconomic index^53^. Combined with the very high proportion of participants reporting white ethnicity, it is not possible to generalise these findings to a broader population. Similarly, when compared to the general UK population, the mean BMI of UK Biobank participants aged 55–64 years was 0.6 and 0.7 lower in men and women respectively^53^. As such, the associations we report may differ in populations with higher rates of obesity. As the focus of this study was on midlife, we used MRI measures of brain health rather than diagnoses of cognitive impairment or dementia as outcomes. Although this has the advantage of helping identify potentially mechanistic pathways, the relevance of these pathways to important clinical outcomes is less clear. Finally, wrist worn accelerometers are unable to capture all activity types (for example, swimming and cycling) and may alter the behaviour of the participant as they are visually aware of the device. As such, it is possible that we have not identified all PA for participants during the measurement period and that PA performed during the measurement period may not reflect longer PA patterns.

## Conclusion

In conclusion, greater PA was associated with larger brain volumes across multiple MRI measured brain regions, as well as less WMHV. These associations, in the case of all measured brain volumes apart from GMV, were mediated by but also remained independent of age, sex, BMI and cardiometabolic risk. Our results support efforts to increase PA to maintain brain health in people in mid-later life, and provide insights into potential pathways involved.

## Electronic supplementary material

Below is the link to the electronic supplementary material.

Supplementary Material 1

## Data Availability

The data that support the findings of this study are available from the UK Biobank but restrictions apply to the availability of these data, which were used under license for the current study, and so are not publicly available. Data are however available from the corresponding authors upon reasonable request and with permission of the UK Biobank (https://www.ukbiobank.ac.uk/enable-your-research/apply-for-access).
