# Supplementary material for: Associations between physical activity and brain structure in a large community cohort
Source: Sci Rep. 2025 May 29;15:18896. doi: 10.1038/s41598-025-04010-7 (PMC12122851; doi:10.1038/s41598-025-04010-7)
Supplement: Supplementary file 1 — Supplementary Material 1 [file 41598_2025_4010_MOESM1_ESM.pdf]

Supplementary Information

Associations between physical activity and brain structure in a large community cohort

Alexandra La Hood [a], Chris Moran [a,b,c,d,e], Stephanie Than [a,b,c,f], Alicia Lu [a,b,c], Taya A Collyer [a,c], Richard Beare [a,c,g], Velandai Srikanth [a,b,c]

[a] Peninsula Clinical School, School of Translational Medicine, Monash University, PO Box 52, Frankston VIC 3199, Australia

[b] Department of Geriatric Medicine, Peninsula Health, 24 Separation Street Mornington VIC 3931, Australia

[c] National Centre for Healthy Ageing, PO Box 52, Frankston VIC 3199, Australia

[d] School of Public Health and Preventive Medicine, Monash University, 553 St Kilda Road, Melbourne 3004, Victoria, Australia

[e] Department of Home, Acute and Community, Alfred Health, 260 Kooyong Rd, Caulfield VIC 3162, Australia

[f] Department of Geriatric Medicine, Western Health, 160 Gordon Street, Footscray VIC 3011, Australia

[g] Developmental Imaging, Murdoch Children's Research Institute, Melbourne, 50 Flemington Rd, Parkville VIC 3052, Australia

Author email addresses

Alexandra La Hood: alexandra.lahood@monash.edu

Chris Moran: chris.moran@monash.edu

Stephanie Than: stephanie.than@wh.org.au

Alicia Lu: alicia.lu@monash.edu

Taya Collyer: Taya.Collyer@monash.edu

Richard Beare: richard.beare@monash.edu

Velandai Srikanth: velandai.srikanth@monash.edu

Corresponding author: chris.moran@monash.edu

Supplementary Table S1 Differences in the associations between physical activity and brain volumes across body mass index strata

	**Total Brain Volume**[a] β-coefficient (p-value)	**Grey Matter Volume**[a] β-coefficient (p-value)	**White Matter Volume** β-coefficient (p-value)	**Total Hippocampal Volume**[a] β-coefficient (p-value)	**White Matter Hyperintensity volume**[a,b] β-coefficient (p-value)
**Model 1** (adjusted for age and sex)					
Body mass index (kg/m^2)					
<20	0.21 (0.41)	0.21 (0.18)	-0.008 (0.96)	0.001 (0.75)	-1×10^{-6} (0.75)
20-25	Reference	Reference	Reference	Reference	Reference
25-30	0.05 (0.69)	0.05 (0.55)	0.03 (0.72)	0.001 (0.54)	8×10^{-7} (0.66)
>30	-0.03 (0.87)	0.17 (0.14)	-0.15 (0.22)	-0.004 (0.25)	-2×10^{-6} (0.59)
**Model 2 (**fully adjusted)					
Body mass index (kg/m^2)					
<20	0.02 (0.94)	0.15 (0.36)	-0.13 (0.44)	-0.0004 (0.93)	-3×10^{-6} (0.41)
20-25	Reference	Reference	Reference	Reference	Reference
25-30	0.09 (0.51)	0.09 (0.29)	0.03 (0.72)	0.002 (0.38)	-5×10^{-7} (0.80)
>30	-0.20 (0.33)	0.04 (0.77)	-0.19 (0.14)	-0.005 (0.17)	-2×10^{-7} (0.94)

[a]Models contain quadratic and cubic physical activity terms; [b]Log-transformed [c]Fully adjusted model includes ethnicity + Townsend deprivation index + hypertension + ischaemic heart disease + hypercholesterolaemia + stroke + diabetes + Apolipoprotein E ε4 + smoking + education + sleep + alcohol + mood

Supplementary Table S2 Influence of waist circumference on associations between physical activity and brain volumes

	**Total Brain Volume**[a]<br><br>β-coefficient<br>(95% CI)<br>p-value	**Grey Matter Volume**[a]<br><br>β-coefficient<br>(95% CI)<br>p-value	**White Matter Volume**<br><br>β-coefficient<br>(95% CI)<br>p-value	**Total Hippocampal Volume**[a]<br><br>β-coefficient<br>(95% CI)<br>p-value	**White Matter Hyperintensity volume**[a,b]<br><br>β-coefficient<br>(95% CI)<br>p-value
**Model 1**					
Physical activity	3.67<br>(2.82 to 4.51)<br>$< 2\times10^{-16}$	3.12<br>(2.57 to 3.67)<br>$< 2\times10^{-16}$	0.43<br>(0.36 to 0.51)<br>$< 2\times10^{-16}$	0.05<br>(0.03 to 0.06)<br>1×10^{-11}	-4.6×10^{-5}<br>(-6×10^{-5} to -3×10^{-5})<br>3×10^{-15}
**Model 2**					
Physical activity + (age × sex)	1.46<br>(0.77 to 2.15)<br>3.4×10^{-5}	1.19<br>(0.77 to 1.60)<br>2.0×10^{-8}	0.11<br>(0.03 to 0.18)<br>0.004	0.015<br>(0.002 to 0.03)<br>0.02	-2.6×10^{-5}<br>(-4×10^{-5} to -2×10^{-5})<br>3×10^{-7}
**Model 3**					
Physical activity + waist + (age × sex)	0.95<br>(0.25 to 1.65)<br>0.008	0.71<br>(0.30 to 1.13)<br>0.0008	0.10<br>(0.02 to 0.17)<br>0.01	0.009<br>(-0.004 to 0.02)<br>0.17	-1.6×10^{-5}<br>(-3×10^{-5} to -6×10^{-6})<br>0.002
Change in Beta compared with Model 2	↓35%	↓40%	↓9%	↓40%	↓38%

[a]Models contain quadratic and cubic physical activity terms; [b]Log-transformed

Supplementary Table S3 Associations between general intensity of physical activity and brain volumes[a]

	**Total Brain Volume** β-coefficient (p-value)	**Grey Matter Volume** β-coefficient (p-value)	**White Matter Volume** β-coefficient (p-value)	**Total Hippocampal Volume** β-coefficient (p-value)	**White Matter Hyperintensity volume[b]** β-coefficient (p-value)
Sedentary (n=16069)	Reference	Reference	Reference	Reference	Reference
Light (n=653)	3.78 (0.11)	2.5 (0.08)	1.26 (0.42)	0.12 (0.003)	-9×10^{-5} (0.01)
Moderate (n=3)	-6.78 (0.84)	-10.4 (0.61)	3.6 (0.87)	0.64 (0.29)	1.7×10^{-4} (0.72)

[a] All models adjusted for age × sex. [b]Log-transformed

Supplementary Table S4 Objectively measured physical activity and brain volumes by self-reported physical activity

	**Low self-reported physical activity**<br><br>Median (IQR)	**Moderate self-reported physical activity**<br><br>Median (IQR)	**High self-reported physical activity**<br><br>Median (IQR)
n	2598	5983	5738
Accelerometer (m*g*)	25.1 (9.09)	27.1 (9.34)	29.6 (11.0)
**Brain volumes (cm^3)**			
Total brain volume	1498 (100)	1495 (103)	1494 (102)
Grey matter volume	795 (64.7)	794 (65.3)	791 (65.4)
White matter volume	702 (55.9)	701 (57.1)	702 (54.7)
Total hippocampal volume	9.99 (1.50)	9.99 (1.48)	9.97 (1.48)
White matter hyperintensities	3.59 (5.15)	3.58 (5.34)	3.70 (5.35)

Supplementary Table S5 Associations between self-reported physical activity and brain volumes

	**Total Brain Volume** β-coefficient (p-value)	**Grey Matter Volume** β-coefficient (p-value)	**White Matter Volume** β-coefficient (p-value)	**Total Hippocampal Volume** β-coefficient (p-value)	**White Matter Hyperintensity volume**[a] β-coefficient (p-value)
**Model 1** (univariable)					
Low	Reference	Reference	Reference	Reference	Reference
Moderate	-3.7 (0.03)	-1.31 (0.25)	-2.42 (0.02)	-0.02 (0.43)	2.5×10^{-6} (0.92)
High	-3.86 (0.03)	-2.75 (0.02)	-1.11 (0.26)	-0.02 (0.47)	9.9×10^{-6} (0.68)
**Model 2 (**Physical activity + (age × sex))					
Low	Reference	Reference	Reference	Reference	Reference
Moderate	-0.80 (0.57)	0.2 (0.81)	-1.00 (0.27)	-0.01 (0.61)	-4.0×10^{-5} (0.05)
High	1.22 (0.39)	0.73 (0.39)	0.49 (0.60)	0.02 (0.44)	-5.1×10^{-5} (0.01)

[a]Log-transformed
